# Aiding food security and sustainability efforts through graph neural network-based consumer food ingredient detection and substitution

**DOI:** 10.1038/s41598-023-44859-0

**Published:** 2023-11-01

**Authors:** Jack Foster, Alexandra Brintrup

**Affiliations:** Department of Engineering, University of Cambridge, Cambridge, CB3 0FS UK

**Keywords:** Environmental impact, Environmental impact, Climate-change policy, Computer science, Statistics

## Abstract

Understanding precisely what is in food products is not always straightforward due to food fraud, differing labelling regulations, naming inconsistencies and the hierarchical nature of ingredients. Despite this, the need to detect and substitute ingredients in consumer food products is far-reaching. The cultivation and production of many ingredients is unsustainable, and can lead to widespread deforestation and biodiversity loss. Understanding the presence and replaceability of these ingredients is an important step in reducing their use. Furthermore, certain ingredients are critical to consumer food products, and identifying these ingredients and evaluating supply-chain resilience in the event of losing access to them is vital for food security analysis. To address these issues, we first present a novel machine learning approach for detecting the presence of unlabelled ingredients. We then characterise the unsolved problem of proposing viable food substitutions as a directed link prediction task and solve it with a graph neural network (GNN).

## Introduction

The negative implications of the wide-spread usage of unsustainable ingredients necessitates a radical reduction in their inclusion in consumer products^1^. One possible method of enacting such change is by applying societal and economic pressure to large manufacturers by empowering ethical consumers to boycott products that contain unsustainable ingredients^2^. In order to apply financial pressure on food businesses, consumers must understand what ingredients are in food products and what sustainable alternatives exist. However, this is non-trivial as food labelling regulations differ globally^3^, food fraud and the mislabelling of ingredients is common^4^, and ingredients are often hierarchical (e.g. peanut butter is often a listed ingredient, but peanut butter itself contains palm oil which may or may not be listed on the product label). In addition, we found that in our dataset of food products sold in the United States, an ingredient can be found under many different names (over 200, in the case of palm oil). Furthermore, consumers often do not understand nutrition labels^5,6^, partly due to there being too much information on the label^6–8^. Even where consumers are knowledgeable about the presence of ingredients in their products, they may still find it hard to apply financial pressure onto organizations as certain ingredients, such as palm oil, are used so broadly^2^. Therefore, manually finding sustainable alternatives may be challenging. The introduction of a data-driven approach to predicting the presence of ingredients, and predicting viable substitutions of unsustainable products, may help overcome some of these barriers. For example, if an ingredient is excluded from a label due to food fraud, then a data-driven approach can identify the product as a statistical anomaly, and predict that it is in fact likely to contain the excluded ingredient.

In addition to avoiding unsustainable products, detecting and substituting ingredients may also be a powerful tool for helping policy-makers and agri-food stakeholders evaluate food security. The food supply-chain is expected to be under growing strain over the coming decades due to challenges such as worker shortages, climate change, and population growth^9^. It is thus necessary to improve supply-chain resilience in order to ensure future food security and availability of consumer products^10^. The Grocery Manufacturers Association estimate that food fraud affects up to $10\%$ of food products globally, costing between $\$10$bn$-\$15$bn^4^. Furthermore, most food fraud cases involve the substitution of a high-value product with a less expensive or lower quality alternative^4^. As a result of food fraud, hierarchical ingredients, differing labelling regulations and naming variations, many food products have hidden dependencies, where ingredients used are not apparent to consumers or to policy makers. Hidden dependencies are a major barrier to allowing policy makers to plan for potential disruptions to the supply-chain^11^. The data-driven approaches presented in this work have direct application to challenges in food security. The first approach presented is able to predict ingredient dependencies, thereby allowing policy makers to better prepare contingency plans in the event of food supply disruptions^12^ and ensure continued, reliable access to safe and affordable food. Furthermore, we present a second method that is capable of identifying viable product substitutions to avoid relying upon specific ingredients. We postulate that understanding how critical an ingredient is (i.e. how often a product is used and how replaceable it is) has significant value to food security analysis and food supply-chain planning processes, as ingredients that are easily replaceable pose lower risk to the food supply-chain than those whose presence is vital. These challenges have been highlighted by food shortages as a result of recent global events such as COVID-19^13^, the United Kingdom leaving the European Union^14^, and the Russian invasion of Ukraine^15^.

A good case study to highlight these issues is palm oil. Palm oil is an edible vegetable oil that is used in a wide range of products not only in the food industry, but also as a biofuel and in pharmaceuticals^16,17^. Oil-palm tree plantations are responsible for large-scale deforestation and massive biodiversity loss across many tropical regions^18–22^, to the extent that oil-palm agriculture was described as the greatest immediate threat to biodiversity in Southeast Asia^2^. In addition, there is mounting evidence that many oil palm plantations utilise labour exploitation practices including forced labour, child labour and sub-minimum wage pay, as well as being guilty of gender discrimination and the use of unsafe working environments^23,24^. As such, research suggests that there is a strong desire from consumers to reduce their intake of palm-oil products^23^. Despite the associated ethical issues, palm oil is widely used and is the most produced vegetable oil globally^25,26^. However, 86% of all palm oil is sourced from just 2 countries, Indonesia and Malaysia^27,28^; this poses a potential risk to many supply-chains, as difficulties in producing or exporting palm oil in these regions could result in significant product shortage globally. These issues illustrate that from both a sustainability and food security perspective, there is a clear incentive to identify the presence and substitutability of palm oil in food products.

To help address the challenges outlined above, this work introduces novel graph neural network-based methods for predicting whether an ingredient is present in a food product and, subsequently, for finding like-for-like product substitutions that avoid the ingredient. Both approaches utilise a graph of food products. For ingredient prediction, products that are made by the same brand are connected by links. We chose this topology as we hypothesise brands may have trends in their ingredient usage. For example, brands may occupy a specific market niche (e.g. brands with a portfolio of confectionery goods may be more likely to release a new chocolate bar than a pasta sauce) and so likely utilise similar ingredients across multiple products. Furthermore, a brand that makes sustainability pledges may be more likely to avoid certain ingredients across all of their products. In contrast, the product substitution task is framed as a link-prediction problem, where directed links between product nodes represent viable substitutions.

Prior to this work, the problems of ingredients prediction and product substitution were largely unsolved. There is, however, some previous work on recipe recommendation, which concerns methods that predict the quality of, or similarity between, home-cooked recipes. These methods typically rely on expert knowledge, such as user comments recommending ingredient substitutions in recipes^29^, user histories informing new recipe recommendations^30^, or food diaries informing food substitutions for meals^31^. Reliance on expert knowledge inhibits the generalisation of models^32^, therefore data-driven methods may learn a greater number of substitutions. Furthermore, viable substitutions are often defined by pairwise similarity of ingredients^29,33^. This metric is too naïve for our purposes as our goal is to avoid ingredients, and maximising the pairwise similarity would mean all ingredients in the products were identical, thus pairwise similarity is an inappropriate metric. In^34^, latent embeddings and a single dense layer are used to predict the presence of an ingredient in a recipe and create “pseudo-recipes” that are used to recommend real recipes that have similar nutrient profiles. While ingredients prediction is performed here, it does not address the challenges posed by hierarchical ingredients. Finally, Graph neural networks have been used to integrate ingredient relationships including co-occurrence and class membership, for example ‘Shiitake $\xrightarrow []{\text {is a}}$ Mushroom’^35^. This work highlights the efficacy of using graph structures for ingredient-based tasks, however, this work is distinct from ours as it did not concern consumer food products, and instead of proposing ingredient conditioned substitutions, the contribution of this paper was the ability to recognise ingredients within food images, which does not solve any of the problems outlined in our work.

None of the methods reviewed solve the challenge of substituting foods to avoid specific ingredients. Furthermore, many of these methods are focused on homemade recipes and not consumer food products, and mostly rely upon expert or auxiliary knowledge to predict either the similarity between foods/recipes or their relative popularity. In the work presented here, we emphasise the importance of using only minimal, readily-available data, such that the method is more accessible and extensible. We thus opt to use information available on product packaging alone, ensuring easier generalisability to other food products and allowing consumers to add to the database of foods without requiring excessive time investment on their part. Next, we outline the methodology for the key contributions of this paper: graph neural network-based ingredients prediction and product substitution methods.

## Results

For both food substitution and ingredients prediction, models were trained to predict the presence of milk, egg, wheat, soy, and palm oil; the results of these were then averaged to produce the results presented here. As model performance is influenced by the target ingredient, these ingredients were chosen a priori with no knowledge of how the models would perform. These specific ingredients were selected as they are commonly used in a range of cuisines, have multiple functionalities, and have reasons for which they may need to be substituted: milk is becoming increasingly relied upon as a food source and thus requires diversification^36,37^, soybean production is a factor in deforestation^38^, climate change poses a serious risk to wheat production^39^, and palm oil is environmentally harmful. In addition egg, milk, wheat and soy are common allergens.

A key challenge that became apparent in our dataset was the presence of hierarchical ingredients, where an ingredient listed on a product is actually composed of multiple ingredients itself (e.g. peanut butter itself contains peanuts, palm oil, salt, and sweeteners), however the product does not necessarily list these constituent ingredients on the label. To that end, our results evaluate model efficacy on two different datasets: “decomposed” and “non-decomposed”. The decomposed dataset is an easier problem, explicitly deconstructing these hierarchies and providing the constituent ingredients as features to the models (although the target ingredient remains masked). In contrast, the non-decomposed data does not provide this additional information, hierarchies are decomposed to acquire accurate labels, but the constituent ingredients are not provided to the model as input. Furthermore, while both datasets feature a test set of unseen products to evaluate generalisation, the non-decomposed test-set is comprised exclusively of products from unseen brands, meaning that this dataset better evaluates the ability of models to generalise without manual decomposition of ingredients, or a familiarity of the brand being evaluated. Evaluating how model performance changes under these constraints is useful in determining how well it may generalise to real-world problems. Finally, we note that for both experiments any ingredients that are present in the test-set but not the train-set are simply ignored and predictions are made from the set of ingredients the models trained against in the training dataset.

For both experiments, the test set accuracy and the area under the ROC curve were used to evaluate model performance. Test-set accuracy is a conventional measure of what percentage of previously unseen samples are correctly classified, whereas AUC is the probability that the model ranks a random positive sample higher than a random negative sample^40^.

### Ingredient prediction

The general methodology employed in this experiment was to present models the ingredients list for a given product, or a graph of products for GNN methods. The model’s objective is to classify products according to whether they likely contain a target ingredient (e.g. palm oil). For products that did contain the target ingredient, this was masked in the model input. This allows the model to learn to infer an ingredient’s presence without requiring it to be explicitly present in the input. For example, when presented with the ingredients of a popular American candy bar:

*{Vanillin, Vegetable Oil, Peanut, Chocolate, Salt, Flavoring, Sugar, Milk, Whey, Soy Lecithin, Cocoa Butter, Cocoa, Emulsifier}*

the graph neural network model correctly predicted that this product did in fact also contain palm oil despite it being masked. A more extensive analysis of the methodology employed is available in section “Materials and methods”. We find that data-driven methods perform well, offering useful predictive power for consumers and policy-makers alike. The ability to make accurate ingredient predictions with incomplete data, in an autonomous way, allows these methods to support key decision making processes in supply-chain resilience and food sustainability challenges. The masking of the target ingredient reduces the reliance on consistent, accurate product labelling. Below, we quantitatively evaluate method performance.

**Figure. 1:**
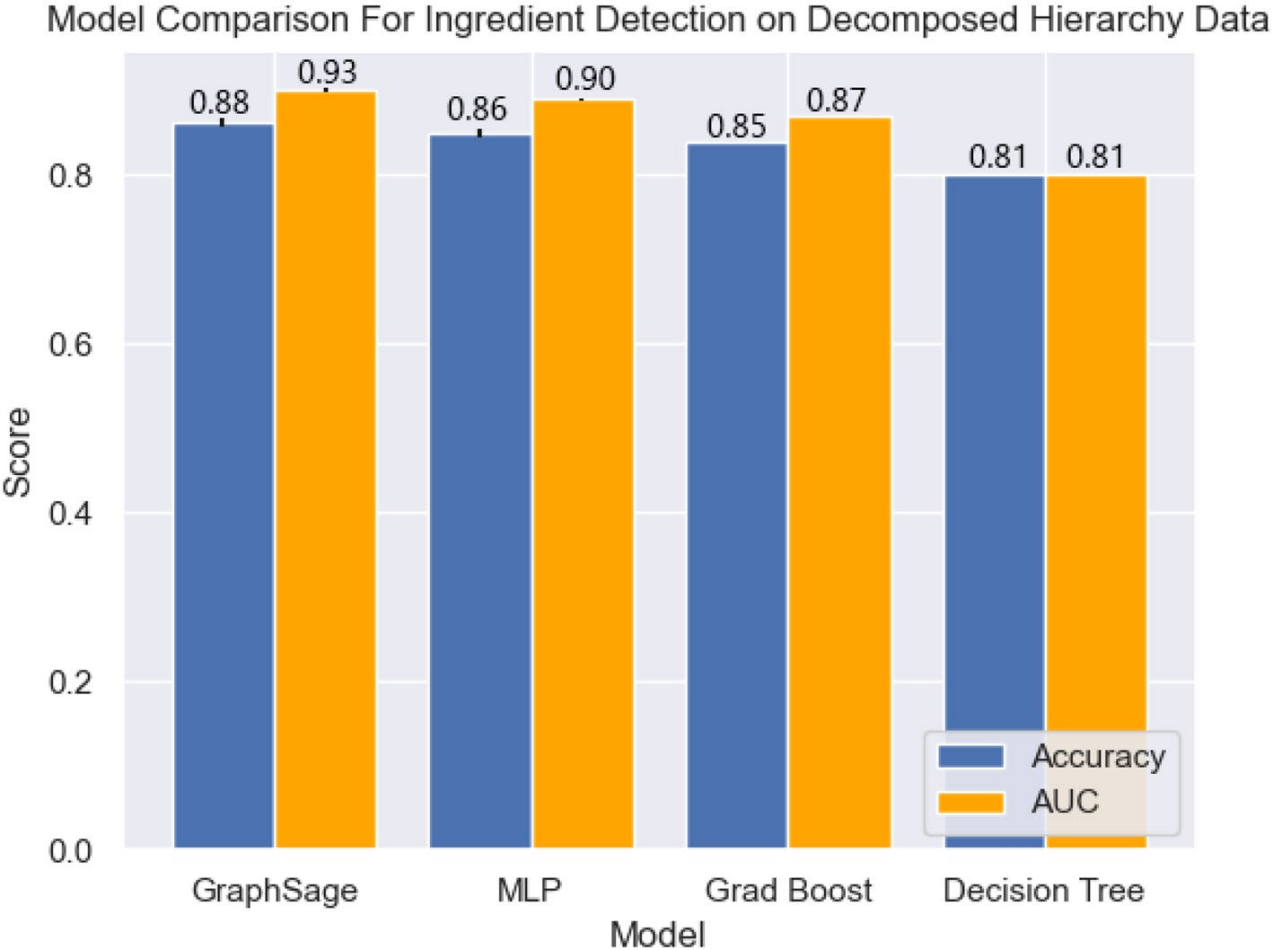
Mean model performance comparison for ingredients prediction on decomposed data, error bars represent one standard deviation.

**Figure. 2:**
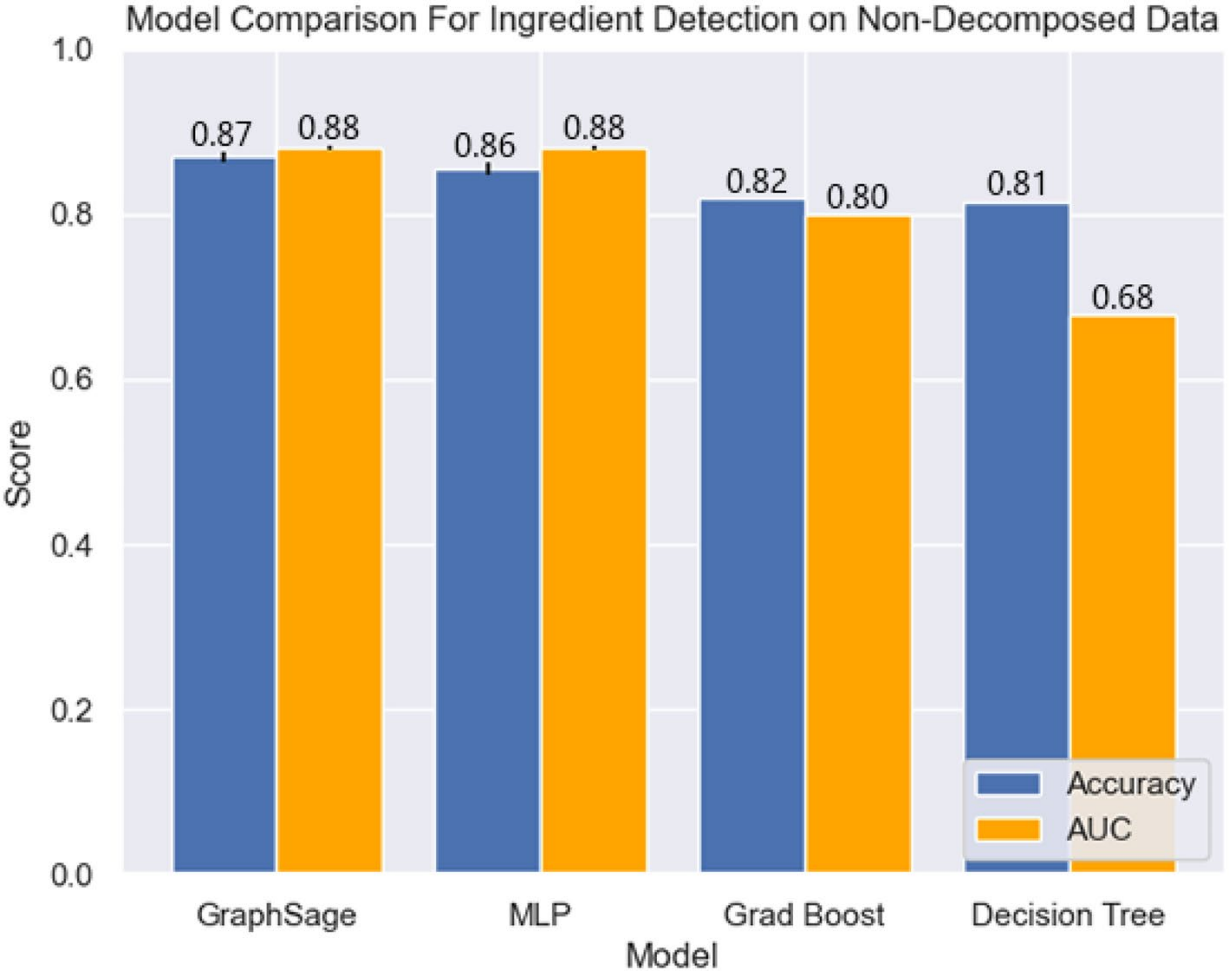
Mean model performance comparison for ingredients prediction on non-decomposed data, error bars represent one standard deviation.

As seen in Figs. 1 and 2, all models are able to complete the ingredients prediction task to a reasonably strong level, all achieving over $80\%$ prediction accuracy and an AUC of at least 0.80. When evaluated on the more challenging non-decomposed test-set, gradient boosting lost $3\%$ accuracy and $0.07\%$ AUC, and the decision tree lost $\sim 0.15$ AUC. This indicates that these methods were less robust to real-world noise in the data, raising concerns over their generalisation ability.

In contrast, neural network-based approaches demonstrated superior performance across the board, especially when transferring to the harder test-set. Both methods performed respectfully, both achieving an AUC of 0.88 on the non-decomposed test set, however the GraphSage model proves superior as it achieves an $87\%$ accuracy on this test set, compared to the $85.5\%$ accuracy of the MLP. The methods performed comparably, indicating that perhaps the brand information was far less important to prediction than that of the ingredients; as there is no clear advantage for the GraphSage model when predicting solely from ingredients, this is one explanation as to why performance was only slightly better - brand information may only be useful in certain fringe cases. One reason for this may be that as the brand vector is sparse, the MLP may be learning to pay less attention to those input features in favour of the more useful ingredients data - thus the introduction of new brands is not as deleterious as it otherwise may have been. Nevertheless, leveraging a graphical structure does yield superior performance. In summary, given the relatively close performance between the GNN and MLP, it is likely that the individual node features are very rich with information, while the graph structure serves a complementary role, augmenting the predictions garnered from the node feature vectors.

### Food substitution

The objective of this experiment was for a graph neural network to identify viable product substitutions. Given a target ingredient, the model had to identify substitutions that satisfied the following criteria:

- The source node product contains the problematic ingredient
- The target node product does not contain said ingredient
- The source and target node share a category (e.g. both are defined as crisps, chocolate bars etc.)
- The cost of both products are within $\pm \,10\%$ of each other

As in ingredient prediction, the target ingredient was masked in the input vector. Given the several conditions that define a valid link in this problem, and the simplicity of the input vector, there was an expectation that obtaining a strong model performance may be challenging. However, when presented with the graph of products, the graph neural network model was able to successfully identify viable and realistic product substitutions and achieve strong performance. For example, within the graph there was an apple-flavored candy product with the ingredients:

*{Corn Syrup, Sugar, Dried Wholemilk, Malic Acid, Whey, Flavoring, Salt, Turmeric Coloring, Soy Lecithin, Blue Coloring, Soy}*

The model successfully identified that this product contained palm oil, before identifying a very similar fruit-flavored candy product. This replacement product filled the same niche, was of similar costs, and used coconut oil rather than the palm oil found in the original product. These features were all implicitly identified by our model from seeing just the ingredients list of the products (excluding palm oil). Further analysis of the methodology is available in section “Materials and methods”.

We believe the potential applications of this are substantial and numerous, such as allowing consumers to avoid ingredients they object to purchasing, or allowing policy-makers to evaluate how reliant sections of the food supply-chain is on specific ingredients. Next, we conduct further quantitative evaluations of the link prediction method.

**Figure. 3:**
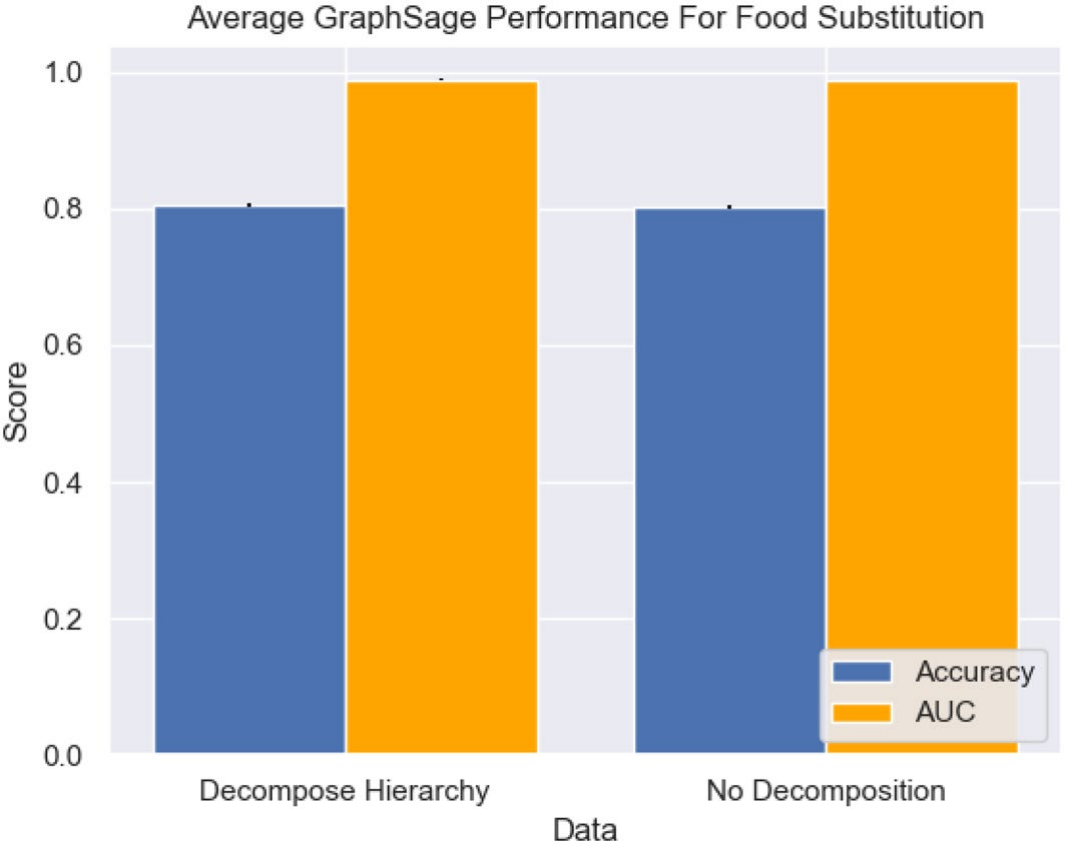
Mean model performance comparison for food substitution on both testing datasets, error bars represent one standard deviation.

Figure 3 shows that accuracy on both test datasets is over $80\%$, and AUC performance is equally impressive achieving over 0.98 on both datasets. The performance drop-off between datasets is negligible, with accuracy dropping from 80.5 to $80.2\%$ which demonstrates that our methods are not only effective at solving the task of ingredients-conditioned product substitution, but also that they are in fact well suited to overcoming the challenges posed by hierarchical ingredients, sparse brand vectors, and labelling variations.

**Figure. 4:**
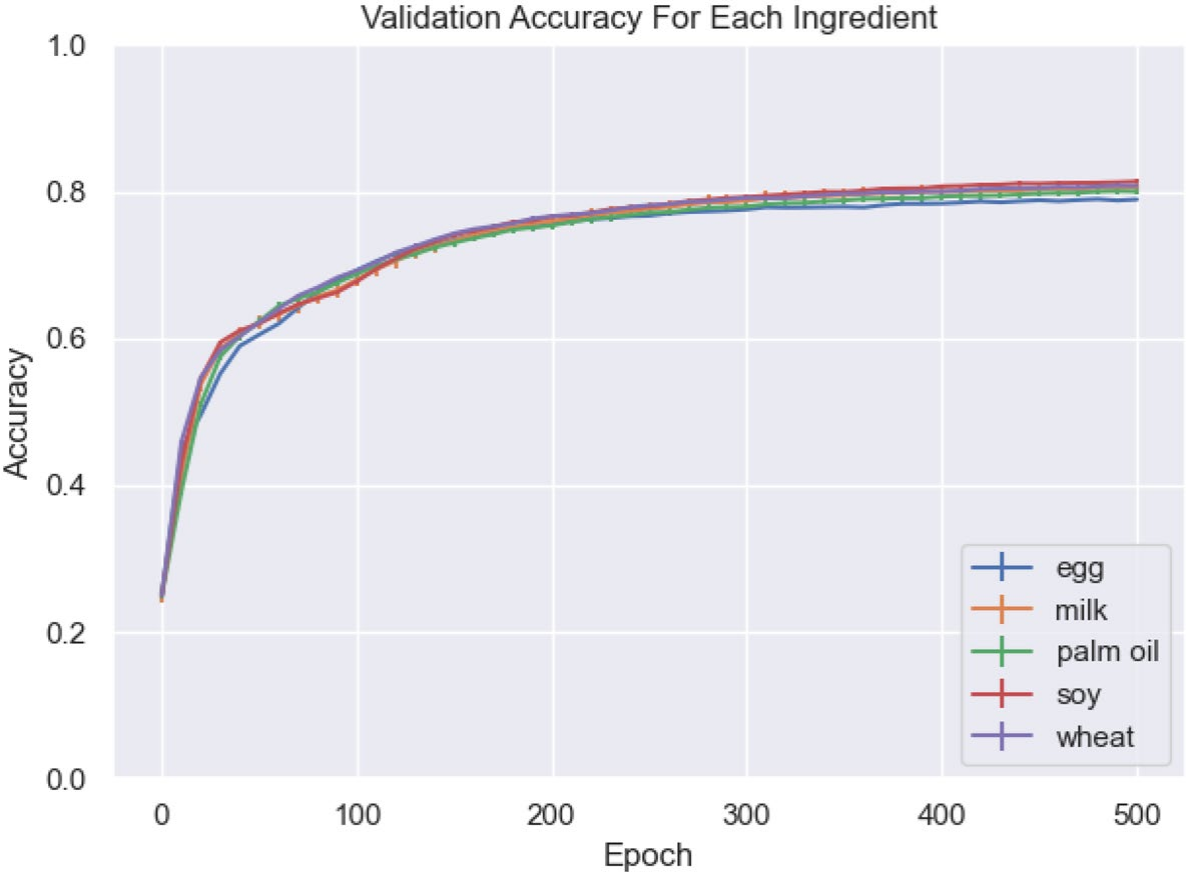
Mean model performance comparison for food substitution, error bars represent one standard deviation.

Figure 4 shows the GraphSage validation accuracy per epoch, for all 5 ingredients evaluated. The model’s performance is consistent for all, dropping below $80\%$ accuracy for only the egg substitutions, and even then still achieving $78.9\%$ accuracy. The ability to maintain performance across different food types is also a promising sign, as this indicates that the method is generalisable and could be used to solve the substitution challenge for many ingredients.

### Limitations

While the performance of the methods presented is impressive, they are not without their limitations. A key limitation of data-driven approaches, and by extension the GNN approach presented here, is that they are inherently probabilistic and are prone to making the occasional mistake. While a probabilistic approach is necessary due to the stochastic and partially observable nature of the problem presented in this work, this has ramifications for their applicability to safety-critical challenges as taking action based on probabilistic models is inherently risky. This problem is compounded by the blackbox nature of neural networks, as their decisions are not explainable or human interpretable. As such, it is recommended that the approaches proposed in this work are used in conjunction with human experts, being utilised as a helpful tool to identify important areas of interest in the food supply-chain, but actions taken based on this insight should be validated by human experts.

The experiments presented here also have their own limitations. Firstly, due to the scarcity of real-world data, only one dataset has been used to evaluate our method. This limits the extent to which generalisation can be analysed. For example, food categories may vary based on region but this cannot be addressed due to data availability. Similarly, model performance is closely tied to the the accuracy of the data; a problem which can be minimised when multiple datasets are available. Furthermore, it is possible that the negative samples generated as part of the link prediction problem are not representative of real-world data. Nevertheless, the negative samples have been generated based on existing precedent set in^41^. Despite these limitations, we feel these methods offer a strong functionality that could be useful in a wide range of food security and sustainability tasks.

Finally, we discuss the endpoints of this work. The methods presented here provide key insight into whether a product is likely to contain an ingredient, as well as whether that product could be substituted for a like-for-like replacement that doesn’t contain the ingredient of interest. While this could help identify ingredients that would have significant impact on the supply-chain, it does not help identify which products are at risk of becoming scarce. For example, there is no analysis of whether an ingredient has diversified sources, or whether the source locations are particularly turbulent (e.g. whether they are at risk of climate change), or indeed whether an ingredient is sourced sustainably. While the method is still of significant value, extending it to identify these characteristics could be extremely valuable and provide an end-to-end pipeline of automated food security and sustainability analysis.

## Discussion

There are many reasons why one may need to detect food ingredients and find substitutes for consumer food products. Understanding and solving these challenges is critical in reducing the use of unsustainable ingredients, as well as to evaluate food security. Allowing a nation to identify weak links and critical ingredients in products can allow preparations to be made to improve their resilience to food shortages. To help alleviate these issues, it is necessary to know exactly what ingredients are in consumer food products and to what extent ingredients are critical to a food category or whether alternatives exist.

Ingredient-conditioned food substitution was previously unsolved, despite its significant practical benefits. By addressing this problem, consumers will be empowered to better understand what is in their food, allowing them to avoid unsustainable or unhealthy ingredients, and apply pressure on food manufacturers to operate in a more sustainable manner. Our work offers a means for policy makers and experts to quickly and autonomously evaluate the criticality of ingredients within consumer foods, and evaluate food security and how robust a country is to disruptions that are common within the global food supply-chain. Manual or rule-based solutions to this problem are limited due to large, complex data, hierarchical food relationships, and labelling variations. In this work, this challenge was solved with a data driven approach, leveraging the merits of graph structure to characterise the novel problem as a directed link prediction task, and solving it with a graph neural network, achieving an accuracy of over $80\%$. Framing the challenge of food substitution as a link prediction task offers a paradigm which is not only efficacious but extensible to new products, making it an ideal framework to apply to real-world scenarios.

Ingredients prediction, while a simpler problem, was also addressed through data-driven means, and we presented four data-driven approaches that could solve the task to varying degrees.

We evaluate substitutions at the product level for several reasons. First, consumers have no ability to change ingredients in existing food products, and instead can contribute to global sustainability efforts by purchasing more sustainable foods and applying financial pressures; providing alternative products is useful here. Second, the role an ingredient plays in a food is highly complex, it may be used for structure, flavor, texture, oxidization, and many other functions^42^. Thus, knowing how to substitute an ingredient is a complex food science problem, and may often require multiple ingredients to replace an existing ingredient^43^. Finally, product-level substitutions offer strong functionality for policy-makers and organizations trying to evaluate supply-chain resilience. The existence of like-for-like substitutions suggests a more resilient supply-chain system where sources for products may be sought, which is a crucial part of ensuring supply-chain resilience^44^. Identifying risks in the supply-chain is increasingly important for building resilience^45^. We propose that the ability to evaluate potential food product substitutions could allow policy makers and organizations to detect high-risk areas of the food supply-chain, where ingredients are overly relied upon, and then conduct what-if scenario planning.

This task was still challenging and unsolved, as no prior work had addressed the labelling variations or hierarchical ingredient problems. Our results show that neural network methods are well suited to overcoming these limitations, and generalising to unseen data. The advantages of graph structure were leveraged, allowing our GNN-method to outperform alternative methods and reach an accuracy of $87\%$. Given the computational cost of GNNs, there is an argument as to whether the relative performance increase from a traditional MLP to a GNN warrants the added computational overhead; for problems where model performance is imperative, the graph approach is best for ingredients prediction. However, for challenges that are less critical, or for those with constrained computational resources, an MLP approach may be better. In this work, we utilised brand data to generate links in our graph, allowing for unseen brands to still offer value (unlike MLP/GradBoost methods). We theorise that the brand information was considerably less important for the prediction task compared to ingredient information, which is why performance gains were not larger. However, this intuition offers a paradigm under which a growing online stream of data may continue to prove useful even as data-drift is experienced, and in future work, we are hopeful that it may be applied to other ingredient prediction problems. For example, in recipe recommendation, links could be between products that are cooked in a similar manner, which may allow for the introduction of new recipes and cooking methods without needing to retrain the model.

Future work in this area could explore several avenues. First, to further increase the application to high-risk and safety critical domains, uncertainty quantification may be used to obtain confidence values for each substitution, allowing more trust to be placed in proposals that are high confidence, and allowing a human-in-the-loop to intervene when the model is uncertain. This would be especially useful for medical applications such as detecting and avoiding allergens. Another avenue could be to introduce real-valued output, such that the directed links have a “substitutability” value. This may be of particular use if no “true” substitution exists, and instead substitutions can be rank-ordered.

We are hopeful that these methods could form the basis of future real world systems, and may be leveraged to reduce the burden on human experts. We envision such systems utilising methods such as these to narrow down the list of candidate substitutions for consumers, or guiding policy maker analysis, rather than relying upon humans trawling through large corpora of possible alternatives.

## Materials and methods

### Data

The dataset used for this work was purchased from the data company Datafiniti, obtained via queries on their data for food and beverages with ingredients from US retailers (Amazon, Walmart and Target)^46^. The data acquired was initially noisy and contained errors. The data was sanitised and processed into a more usable form, resulting in a dataset of $\sim 3000$ products for node classification, and $\sim 3200$ for link prediction. There were 7000 ingredients, and 14394 links in the node classification graph; for link prediction there was some variance in link number due to a different number of substitutions, but for palm oil there were 15,890 links. Preprocessing steps included stemming words to remove suffixes to increase entity matching^47^, removing duplicates and entries with NaN values, and the removal of stop words (such as “the”, along with some additional, food-related stop words such as “contains”). Products had the features: $\{$*name, brand, categories, minimum price, maximum price, ingredients*$\}$. Here, categories are the set of food types to which the product belongs (e.g. crisps, chocolate bar, cereal); a product may belong to multiple categories. The dataset was subsequently split into training, validation, and two different test sets (decomposed and non-decomposed).

For the training, validation and decomposed test set, hierarchical relationships were decomposed, such that if a product listed ‘peanut butter’, for example, then this product would also have common ingredients found in peanut butter added to it (e.g. peanuts, palm oil, etc). These hierarchical ingredients were found by selecting the set of ingredients that were also listed as products within our dataset (e.g. if a product name was *Brand Name Peanut Butter*, then peanut butter would be a hierarchical ingredient). For example, wine products listed in our dataset almost always contained the following ingredients: $\{$‘sugar’, ‘water’, ‘grape’, ‘sulphites’$\}$, and so these could be reasonably inserted into products that listed wine as an ingredient. By cross-checking our ingredients with our products, we are able to significantly reduce the number of hierarchical relationships in our data, however we acknowledge there may exist a small number that have been missed, especially as this required manual verification and therefore does not scale well. For the non-decomposed test set, this process only occurred as a means to check if the target ingredient was contained in the food (e.g. if peanut butter is an ingredient is will most likely contain palm oil). However, this decomposition was not stored or given to the model as input, it was only used to set the target label. In addition, the non-decomposed test-set only contains products from brands not in the train-set, meaning that this dataset better evaluates the ability of models to generalise to new data.

### Development of prediction and substitution models

We propose that manual and rule-based approaches to ingredients prediction and product substitution are undesirable. This is because the scale of the problem, paired with the hidden dependency and name resolution problems discussed previously, results in a prohibitively complex problem that cannot be sufficiently described by a simple set of rules, and manual analysis is a slow process that requires expert domain knowledge^48^. Furthermore, by their very nature there is always a degree of uncertainty around the existence of hidden ingredient dependencies; that is to say, the presence of an ingredient can be defined to be probable, but it often cannot be known for definite whether it exists. We postulate that deep learning methods are well suited to solving the problems of ingredient detection and substitution, as they excel at pattern recognition and are able to generalise to unseen data^32,49^, solving problems without requiring expert domain knowledge or explicit rule definitions. Furthermore, machine learning methods are typically probabilistic and therefore can appropriately manage the uncertainty associated with the tasks^50^.

Graph neural networks utilise permutation-invariant and permutation-equivariant operations which allows the model to utilise the non-Euclidean structural information found within graphs^51^. GNN layers take as input the graph data and output latent embeddings of the node, edge or graph features. For example, a latent node embedding may be generated from a function that considers the features of both the target node and its neighbours. From these embeddings, the model may conduct graph-, node-, and edge-level tasks. In our work here, the GraphSage model was found to be the most effective for this task, likely due to the known efficacy of GraphSAGE on graphs with rich node features^52^ and the added advantage of the GraphSAGE model is that it also facilitates inductive learning, meaning that unlike most other GNN models GraphSAGE can efficiently generalise to unseen nodes.

Neural network models were trained for 400 epochs on the training set and early stopping was applied according to the validation score. The final evaluation of the model was conducted on the test set performance.

For both ingredient prediction and substitution, models were run 25 times to obtain a set of medians and variances that were statistically evaluated. This methodology ensures that the evaluation is statistically robust. This was repeated for 5 ingredients: palm oil, wheat, milk, egg, and soya. The results presented here are the mean performance of the median runs across those five ingredients. It should be noted that for both ingredient prediction and product substitution, the target ingredient (e.g. palm oil) is masked in the feature vectors, to ensure the model cannot see explicitly whether the ingredient is present in the product.

#### Food ingredient prediction

For ingredient prediction, the objective was to train a model capable of predicting whether a product contains a specific ingredient. Achieving this in a data-driven way would help overcome barriers imposed by food fraud and other labelling inconsistencies by identifying hidden ingredient dependencies.

Each product had a binary encoded feature vector, describing what ingredients were present in the food (with the target ingredient masked). For the graph approach, this was framed as a node classification problem. Within the graph, products were connected if they were made by the same brand. Here, the model must predict whether a product does or does not contain the target ingredient using information from the product’s feature vector as well as from connected nodes, giving wider context than alternative methods. For non-graph approaches, the problem is framed as a traditional classification task, where the input vector is a concatenation of a one-hot encoding of the brands and the product’s ingredient vector. From this information, the models predicted whether products were likely to contain a target ingredient. Four different models were evaluated: Decision Tree, Gradient Boosting, Multi-Layer Perceptron (MLP), and a GraphSage GNN. Decision trees were implemented as they are simple and provide a good baseline performance on which other models may build. Gradient tree boosting is used in literature for recipe prediction and user rating prediction^29^, while multi-layer perceptrons are used here in place of the dense single-layer model implemented in previous work^34^. This change is to ensure that the GNN model does not outperform the MLP by simply having vastly more parameters.

The brand-conditioned graph structure allows for inferences to be made about these trends. For example, if at test time a trained model is presented a new set of products from a new brand, an MLP would view each product sequentially and in isolation, with the brand input offering no value as it is previously unseen. In contrast, the GNN will view the data as a graph, with the products from the new brand connected to each other. When this model makes a prediction, its forward pass will apply a convolution over this connected subgraph, thus extracting any useful information that may exist from the neighbouring products that is not possible with an MLP or XGBoost model. Furthermore, a limitation of traditional classification methods compared to the graph approach is that if the model is presented with new data, from previously unseen brands, then the brand portion of the input vector will offer no value. In contrast, as the graph topology is dictated by the brand vector, unseen brands still have value for the GNN approach, as it dictates the nodes over which graph convolutions will be applied^53^.

The decision tree utilised the Gini impurity criterion, which is the likelihood an instance of a random variable will be misclassified^54^. While the decision tree approach is simple and was not expected to perform as well as other methods, it has the added advantage that it is fully transparent and explainable. This is particularly useful for policy applications, where due to the high risk nature of the field it is hard to utilise the blackbox predictions of other machine learning models.

Gradient tree boosting was also implemented^55,56^. Here, 100 boosting stages were used to create an ensemble of weak decision trees, with a maximum depth of 3, and the Friedman mean square error criterion^55^.

The multi-layer perceptron had 128 hidden channels, a dropout of $p=0.5$ and utilised the ReLU activation function. A learning rate of 0.01 and a weight decay of $5e-3$ were used alongside the Adam optimiser^57^, and the cross entropy loss function. As the GraphSAGE model defined links with respect to common brands, the MLP was trained under two different regimes. First, just the ingredients vector was used (as with the decision tree and gradient boosting); however, the MLP was also trained using a concatenated binary encoded vector of the ingredients and brands. This ensured that the GraphSAGE model could be reasonably compared to the MLP, allowing an evaluation of the importance of the graph structure to be made.

As mentioned above, the GraphSAGE GNN model was empirically found to outperform other GNN architectures, including a vanilla graph convolutional network^58^, and a graph attention network (both v1 and v2)^59,60^. The GraphSAGE utilised 128 hidden channels, alongside the same loss function, optimizer, learning rate and weight decay values as the MLP model.

Several different graph structures were implemented for input to the GNN, links were defined as: shared ingredients, shared brand or shared categories. Empirically, it was found that the shared brand graph outperformed the alternative topologies.

#### Food substitution

**Figure. 5:**
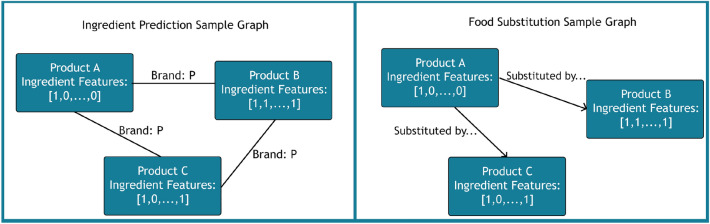
(**a**) Example of the ingredients prediction input graph. Nodes contain binary encoded ingredients vectors, and undirected links exist between products that are made by the same brand. This task involves the binary classification of each node, which equates to the model’s prediction of whether the product contains the specified ingredient. (**b**) Example of the food substitution graph. Nodes contain binary encoded ingredients vectors, and directed links exist from a product to be replaced, to a viable substitution. The model must predict the existence of these links, compared to the existence of negatively (random) links that do not satisfy the substitution criteria.

We frame food substitution as a directed link prediction problem with nodes as food products and directed links representing viable substitutions (Fig. 5b). Product nodes have a feature vector describing the ingredients they contain. A graph approach to this problem is a natural choice, as substitutions are elegantly represented by directed links, a graph model facilitates the existence of multiple substitutions for each product, and finally the graph structure allows a GNN model to utilise known viable substitutions to inform the discovery of new substitutions. Our model predicts the existence of viable substitutions by identifying links that satisfy the following criteria:

- The source node product contains the problematic ingredient
- The target node product does not contain said ingredient
- The source and target node share a category (e.g. both are defined as crisps, chocolate bars etc.)
- The cost of both products are within $\pm 10\%$ of each other

The first two criteria are obvious, naturally the substitution must actually be substituting the target ingredient. In our dataset, the category feature of a product dictates what kind of food it is. For example, *Flatbreads, Cookies*, and *Chocolate* are all categories present in the dataset. Requiring replacement products share a category ensures that substitutions represent like-for-like replacements. Finally, the cost constraint increases the likelihood that substitutions are realistic, equivalent, and will not disrupt consumer’s budgets. Since the model does not have access to category or cost information, these characteristics are inferred entirely from ingredients information alone.

As with ingredients prediction, the node features are a binary encoded vector of a product’s ingredients (with the target ingredient removed). The link prediction is formulated in the manner presented in^41^, where the ground truth edges are calculated and treated as positive samples (i.e. these edges conform to the above constraints), and a set of negative samples are generated by randomly selecting non-existent edges within the graph. These random links are re-sampled at every epoch, and thus over the course of full training the negative set becomes a reasonable approximation of the full set of negative links, while ensuring the model has balanced data to predict at each epoch. The task of the GNN is to then conduct binary classification of the set of links into positive and negative samples (by means of outputting a probability that a given link is valid).

A GraphSAGE model was once again used, this time with 128 hidden channels and 128 out channels. The Adam optimiser was used once more, with a learning rate of 0.001, accompanied by a sigmoid layer and the binary cross entropy loss function.

As the feature vector for each node is the ingredients of the product minus the target ingredient, the model is never explicitly aware of any of the constraints: the presence of the target ingredients is unknown, and the cost and category of the products is not present in the feature vector either. Therefore, in order to successfully solve this link prediction task, all of these properties must be approximated from just the product’s ingredients.

### Supplementary Information

Supplementary Information 1.

Supplementary Information 2.

## Data Availability

All data generated or analysed during this study are included in this published article (and its supplementary information files).

## Code availability

All code is available here https://github.com/jwf40/GNN-Ingredient-Detection-and-Substitution.

## References

[1] Parsons S, Raikova S, Chuck CJ (2020). The viability and desirability of replacing palm oil. Nat. Sustain..

[2] Wilcove DS, Koh LP (2010). Addressing the threats to biodiversity from oil-palm agriculture. Biodivers. Conserv..

[3] Charlebois S, Sterling B, Haratifar S, Naing SK (2014). Comparison of global food traceability regulations and requirements. Compr. Rev. Food Sci. Food Saf..

[4] Johnson, R. Food fraud and economically motivated adulteration of food and food ingredients (2014).

[5] Campos S, Doxey J, Hammond D (2011). Nutrition labels on pre-packaged foods: A systematic review. Public Health Nutr..

[6] Koen N, Blaauw R, Wentzel-Viljoen E (2016). Food and nutrition labelling: The past, present and the way forward. South Afr. J. Clin. Nutr..

[7] Geiger CJ, Wyse BW, Parent CM, Hansen RG (1991). Review of nutrition labeling formats. J. Am. Diet. Assoc..

[8] Cowburn G, Stockley L (2005). Consumer understanding and use of nutrition labelling: A systematic review. Public Health Nutr..

[9] Ambler-Edwards, S. *et al.* Food futures: Rethinking UK strategy. A Chatham house report (2009).

[10] Ponomarov SY, Holcomb MC (2009). Understanding the concept of supply chain resilience. Int. J. Logist. Manag..

[11] Brintrup A (2018). Predicting hidden links in supply networks. Complexity.

[12] Kosasih EE, Brintrup A (2022). A machine learning approach for predicting hidden links in supply chain with graph neural networks. Int. J. Prod. Res..

[13] Sharma HB (2020). Challenges, opportunities, and innovations for effective solid waste management during and post COVID-19 pandemic. Resour. Conserv. Recycl..

[14] Lang T, McKee M (2018). Brexit poses serious threats to the availability and affordability of food in the united kingdom. J. Public Health.

[15] Hellegers P (2022). Food security vulnerability due to trade dependencies on Russia and Ukraine. Food Secur..

[16] Basiron Y (2002). Palm oil and its global supply and demand prospects. Oil Palm Ind. Econ. J..

[17] Azhar B, Saadun N, Prideaux M, Lindenmayer DB (2017). The global palm oil sector must change to save biodiversity and improve food security in the tropics. J. Environ. Manag..

[18] Tan K, Lee K, Mohamed A, Bhatia S (2009). Palm oil: Addressing issues and towards sustainable development. Renew. Sustain. Energy Rev..

[19] Butler RA, Laurance WF (2008). New strategies for conserving tropical forests. Trends Ecol. Evol..

[20] Gutiérrez-Vélez VH (2011). High-yield oil palm expansion spares land at the expense of forests in the Peruvian amazon. Environ. Res. Lett..

[21] Fitzherbert EB (2008). How will oil palm expansion affect biodiversity?. Trends Ecol. Evol..

[22] Santika T (2021). Impact of palm oil sustainability certification on village well-being and poverty in Indonesia. Nat. Sustain..

[23] Sodano V, Riverso R, Scafuto F (2018). Investigating the intention to reduce palm oil consumption. Calitatea.

[24] Amnesty International. *Indonesia: The Great Palm Oil Scandal: Labour abuses behind big brand names: Executive summary* (ASA 21/5243/2016, 2016). www.amnesty.org/en/documents/asa21/5243/2016/en/. Accessed online December 2021.

[25] Mba OI, Dumont M-J, Ngadi M (2015). Palm oil: Processing, characterization and utilization in the food industry—A review. Food Biosci..

[26] Carter C, Finley W, Fry J, Jackson D, Willis L (2007). Palm oil markets and future supply. Eur. J. Lipid Sci. Technol..

[27] Mancini, A. *et al.* Biological and nutritional properties of palm oil and palmitic acid: effects on health. *Molecules* **20**, 17339–17361 (2015). https://www.mdpi.com/1420-3049/20/9/17339/htm. 10.3390/molecules200917339 PMC6331788 26393565

[28] Monzon JP (2021). Fostering a climate-smart intensification for oil palm. Nat. Sustain..

[29] Teng, C.-Y., Lin, Y.-R. & Adamic, L. A. Recipe recommendation using ingredient networks. In *Proceedings of the 4th Annual ACM Web Science Conference*, 298–307 (2012).

[30] Freyne, J. & Berkovsky, S. Recommending food: Reasoning on recipes and ingredients. In *International Conference on User Modeling, Adaptation, and Personalization*, 381–386 (Springer, 2010).

[31] Achananuparp, P. & Weber, I. Extracting food substitutes from food diary via distributional similarity. arXiv:1607.08807 (2016).

[32] Krizhevsky, A., Sutskever, I. & Hinton, G. E. Imagenet classification with deep convolutional neural networks. In *Advances in Neural Information Processing Systems*, 1097–1105 (2012).

[33] Elsweiler, D., Trattner, C. & Harvey, M. Exploiting food choice biases for healthier recipe recommendation. In *Proceedings of the 40th International ACM SIGIR Conference on Research and Development in Information Retrieval*, 575–584 (2017).

[34] Chen M (2020). Eating healthier: Exploring nutrition information for healthier recipe recommendation. Inf. Process. Manag..

[35] Chen J (2020). Zero-shot ingredient recognition by multi-relational graph convolutional network. Proc. AAAI Conf. Artif. Intell..

[36] Herrero M (2015). Livestock and the environment: What have we learned in the past decade?. Annu. Rev. Environ. Resour..

[37] Thornton PK, Herrero M (2015). Adapting to climate change in the mixed crop and livestock farming systems in sub-Saharan Africa. Nat. Clim. Change.

[38] Fehlenberg V (2017). The role of soybean production as an underlying driver of deforestation in the South American Chaco. Glob. Environ. Change.

[39] Porter, J. R. *et al.* Food security and food production systems (2014).

[40] Bradley AP (1997). The use of the area under the roc curve in the evaluation of machine learning algorithms. Pattern Recognit..

[41] Zhang, M. & Chen, Y. Link prediction based on graph neural networks. *Adv. Neural Inf. Process. Syst.* **31** (2018).

[42] Indrani, D. & Rao, G. V. Functions of ingredients in the baking of sweet goods. In *Food Engineering Aspects of Baking Sweet Goods*, 31–47 (2008).

[43] Hinrichsen N (2016). Commercially available alternatives to palm oil. Lipid Technol..

[44] Lin Y, Fan D, Shi X, Fu M (2021). The effects of supply chain diversification during the COVID-19 crisis: Evidence from Chinese manufacturers. Transp. Res. Part E Logist. Transp. Rev..

[45] Katsaliaki K, Galetsi P, Kumar S (2021). Supply chain disruptions and resilience: A major review and future research agenda. Ann. Oper. Res..

[46] Brintrup, A. & Datafiniti. Supermarket food product data from Amazon, Walmart and target (2020). Food and beverage products that contained ingredients were queried via Datafiniti online portal.

[47] Porter MF (1980). An algorithm for suffix stripping. Program.

[48] LeCun, Y., Bengio, Y. & Hinton, G. Deep learning. *Nature* **521**, 436–444 (2015). 10.1038/nature14539 26017442

[49] Hershey, S. *et al.* CNN architectures for large-scale audio classification. In *2017 IEEE International Conference on Acoustics, Speech and Signal Processing (ICASSP)*, 131–135 (IEEE, 2017).

[50] Bishop CM, Nasrabadi NM (2006). Pattern Recognition and Machine Learning.

[51] Wu Z (2020). A comprehensive survey on graph neural networks. IEEE Trans. Neural Netw. Learn. Syst..

[52] Hamilton, W. L., Ying, R. & Leskovec, J. Inductive representation learning on large graphs. In *Proceedings of the 31st International Conference on Neural Information Processing Systems*, 1025–1035 (2017).

[53] Zhou J (2020). Graph neural networks: A review of methods and applications. AI Open.

[54] Xia F, Zhang W, Li F, Yang Y (2008). Ranking with decision tree. Knowl. Inf. Syst..

[55] Friedman, J. H. Greedy function approximation: A gradient boosting machine. *Ann. Stat.* 1189–1232 (2001).

[56] Friedman JH (2002). Stochastic gradient boosting. Comput. Stat. Data Anal..

[57] Kingma, D. P. & Ba, J. Adam: A method for stochastic optimization. arXiv preprint arXiv:1412.6980 (2014).

[58] Kipf, T. N. & Welling, M. Semi-supervised classification with graph convolutional networks. arXiv preprint arXiv:1609.02907 (2016).

[59] Veličković, P. *et al.* Graph attention networks. arXiv preprint arXiv:1710.10903 (2017).

[60] Brody, S., Alon, U. & Yahav, E. How attentive are graph attention networks? arXiv preprint arXiv:2105.14491 (2021).

